# The chromosomal genome sequence of the giant green anemone, *Anthopleura xanthogrammica* (Brandt, 1835) (Actiniaria: Actiniidae)

**DOI:** 10.12688/wellcomeopenres.27544.1

**Published:** 2026-09-01

**Authors:** Samuel Bedgood, Michael N Dawson, Graeme Oatley, Elizabeth Sinclair, Eerik Aunin, Noah Gettle, Camilla Santos, Michael Paulini, Haoyu Niu, Victoria McKenna, Rebecca O’Brien, Wellcome Sanger Institute Tree of Life Management, Samples and Laboratory team, Wellcome Sanger Institute Scientific Operations: Sequencing Operations, Wellcome Sanger Institute Tree of Life Core Informatics team, EBI Aquatic Symbiosis Genomics Data Portal Team, Aquatic Symbiosis Genomics Project Leadership

**Affiliations:** 1 Division of Natural Sciences, New College of Florida, Sarasota, Florida, USA; 2 University of California Merced Dawson Lab, Merced, California, USA; 3 Tree of Life Programme, Wellcome Sanger Institute, Hinxton, England, UK

**Keywords:** Anthopleura xanthogrammica, giant green anemone, genome sequence, chromosomal, Actiniaria

## Abstract

We present a genome assembly from a specimen of *Anthopleura xanthogrammica* (giant green anemone; Cnidaria; Anthozoa; Actiniaria; Actiniidae). The genome sequence has a total length of 290.04 megabases. Most of the assembly (97.36%) is scaffolded into 19 chromosomal pseudomolecules. The mitochondrial genome has also been assembled, with a length of 20.04 kilobases. Gene annotation of this assembly by Ensembl identified 25,034 protein-coding genes.

## Species taxonomy

Eukaryota; Opisthokonta; Metazoa; Eumetazoa; Cnidaria; Anthozoa; Hexacorallia; Actiniaria; Actiniidae; *Anthopleura*; *Anthopleura xanthogrammica* (Brandt, 1835) (NCBI:txid6112).

## Background

**Figure 1.**
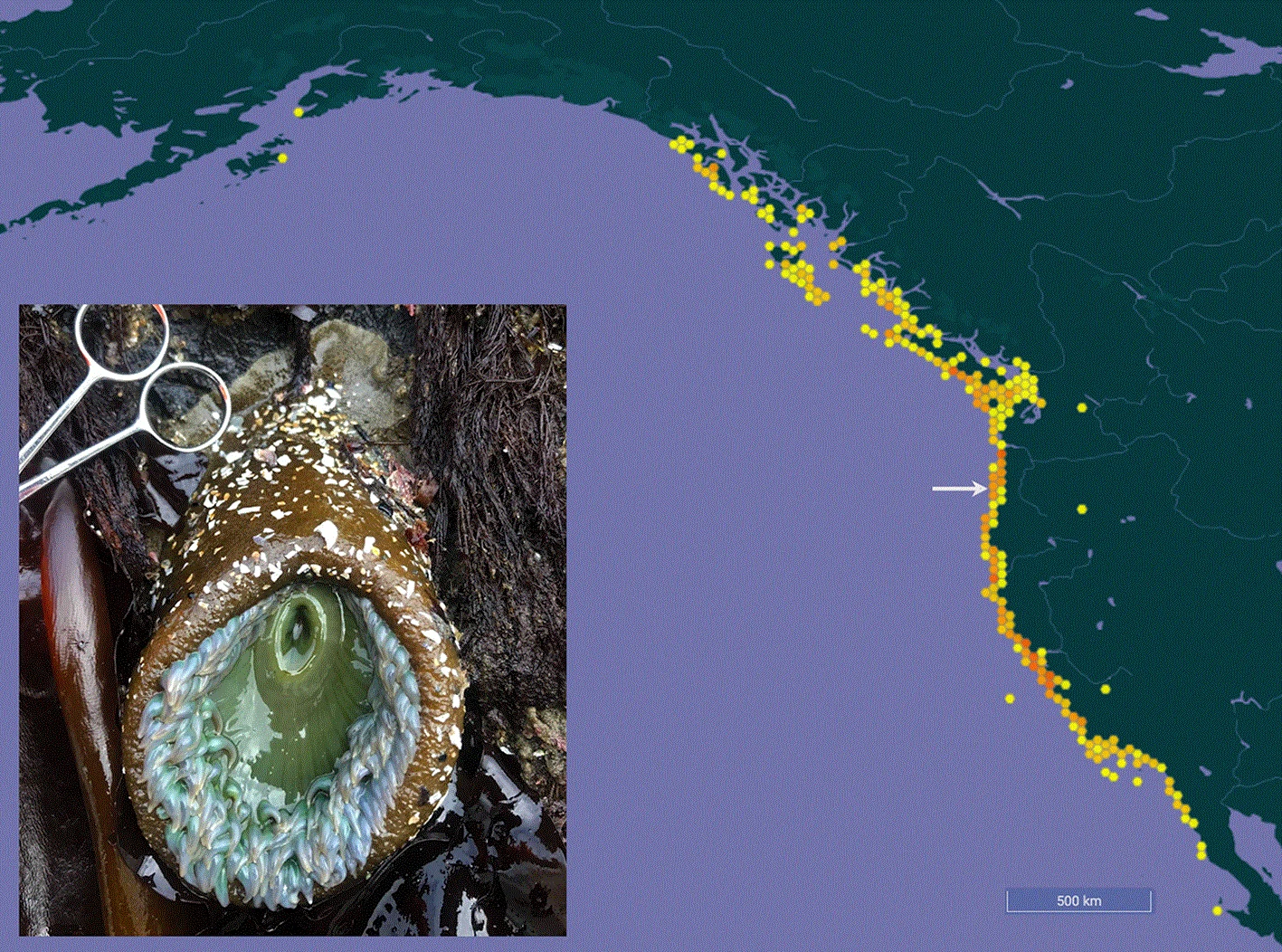
Plate Carrée projection of the northeastern Pacific showing the geographical distribution of *Anthopleura xanthogrammica*, based on observations recorded by GBIF.org from 1960 to the present and downloaded on 30 August 2025. Redder hexagons indicate more observations, which may reflect greater abundance and/or recording effort. Non-coastal points are erroneous; observations at the extremes of the latitudinal range may represent extralimital records or misidentifications. The white arrow indicates the approximate sampling location (latitude 44.4955, longitude −124.0848) of the sea anemone shown in the inset and used for reference genome generation.

*Anthopleura xanthogrammica* is a large (up to 25 cm in diameter and 30 cm tall when extended), primarily intertidal sea anemone of the northeastern Pacific Ocean from Alaska to Panama ( Haderlie *et al.*, 1980; Hand, 1955) ( Figure 1). Its common name “giant green sea anemone” speaks to its spectacular green appearance when unfurled in tidepools ( Figure 1 inset). *A. xanthogrammica*’s oral disk is green or rarely blue with no prominent radial lines on the disk as seen in *A. sola*, one of several congeneric species in the region ( Bedgood, 2021; Pearse & Francis, 2000). The most definitive way to distinguish *A. xanthogrammica* from its congeners is by the spacing of the verrucae, which are bumps on the column of the anemone. *A. xanthogrammica* has densely packed verrucae with little column visible, whereas congeners ( *A. sola* and *A. elegantissima*) have sparse verrucae in rows along the column ( Fautin & Hand, 2007).

Like many symbiotic cnidarians, the colour of *A. xanthogrammica* results from pigments and fluorescent proteins, which provide protection against oxidative stress ( Clarke *et al.*, 2024; Staats *et al.*, 2024). Its golden brown-green or vivid green undertone is produced, respectively, by a symbiotic dinoflagellate in the family Symbiodiniaceae, *Breviolum* ‘muscatinei’ ( Cornwell & Hernández, 2021), or the chlorophyte *Elliptochloris marina* ( Letsch *et al.*, 2009). It is notable that *A. xanthogrammica* can associate with two algal species from different phyla that are sometimes found mixed within individual anemone hosts from northern California to Washington ( Secord & Augustine, 2000). It is not uncommon to also find anemones that are aposymbiotic or without symbionts in habitats covered with sand, shaded, or recently exposed to high temperatures ( Bedgood, 2021). *A. xanthogrammica* is incorporated in the Aquatic Symbiosis Genomics project as a benthic and photosymbiotic reference taxon. Its ability to form symbioses with the dinoflagellate *B.* ‘muscatinei’, the chlorophyte *E. marina*, or both, and to occur naturally in an aposymbiotic form, makes it valuable for studying how environmental conditions, genetics and opportunities for association affect its symbioses.

*A. xanthogrammica* exhibits a mixotrophic feeding strategy. It obtains nutrients both autotrophically and heterotrophically and may switch between strategies according to prey availability ( Bedgood *et al.*, 2020). Its diet includes a variety of intertidal organisms, ranging from fish and crustaceans to significantly larger prey such as jellyfish, sea stars, and even terrestrial vertebrates that enter the intertidal zone (S. Bedgood, pers. obs.). Notably, documented observations indicate that *A. xanthogrammica* has consumed juvenile seabirds that have fallen from their nests ( Guy *et al.*, 2014). Such opportunistic predation highlights the ecological role of *A. xanthogrammica* as both a predator and a scavenger. *A. xanthogrammica* also exerts a non-trophic influence by enhancing the richness of rocky intertidal communities, creating microhabitats that attract mobile invertebrates to the edges of anemones during low tides ( Bedgood *et al.*, 2023).

*A. xanthogrammica* is a broadcast spawner, releasing eggs or sperm into the water column. Once fertilisation occurs, planula larvae form and move through the water column in search of a site on which to settle ( Siebert, 1974). Juveniles are found in mussel beds on rocky intertidal shores ( Sebens, 1981). As they grow, they crawl using their muscular pedal disk and settle into adjacent habitats where there is more space and potentially greater food availability ( Sebens, 1981). Once established, these sea anemones rarely move unless they are attacked by congeners or disturbed ( Sebens, 1982).

A chromosome-scale reference genome has been published for the closely related and geographically overlapping congener *A. sola* ( Cornwell *et al.*, 2022). A chromosome-level genome has been sequenced for *A. artemisia* (GCA_964304775.1). Together, these genome resources provide a reference framework for comparing the ecology of these species, including differences in their microhabitats, distributions and environments. They will also provide opportunities to examine relationships between hosts and photosymbionts, including the occurrence of aposymbiotic forms of *A. xanthogrammica.*

*A. xanthogrammica* is common throughout much of its range and not considered threatened. However, as an abundant and ecologically influential species in rocky shore communities, *A. xanthogrammica* plays a key role in structuring biotic interactions. It serves as a predator, scavenger and competitor for space, provides microhabitats, and hosts algal symbionts. Given its functional importance, shifts in the abundance, physiology, or distribution of *A. xanthogrammica* due to ocean warming, acidification, or other anthropogenic stressors could have cascading impacts on the broader intertidal ecosystem ( Harley *et al.*, 2006; Menge *et al.*, 2008). The availability of a high-quality genome for this species will facilitate mechanistic insights into resilience and vulnerability across biological scales, from individual to ecosystem.

## Methods

### Sample acquisition

Replicate tissue samples containing oral disk, tentacle, and column tissue were excised from the *Anthopleura xanthogrammica* shown in Figure 1 (specimen ID UCALI0000033, ToLID jaAntXant3) at Seal Rock, Oregon USA (44.4955, −124.0848). *Anthopleura xanthogrammica* samples were collected at low tide on 2022-07-15, placed in cryogenic vials, and kept on dry ice until they were stored in a − 80 °C freezer. A second specimen was used for Hi-C sequencing (specimen ID UCALI0000035, ToLID jaAntXant4). It was collected from Hopkins Marine Station, California, USA (latitude 36.6217, longitude 121.9046) on 2022-11-09. The jaAntXant4 specimen was also used for RNA sequencing.

### Nucleic acid extraction

Protocols for high molecular weight (HMW) DNA extraction developed at the Wellcome Sanger Institute (WSI) Tree of Life Core Laboratory are available on protocols.io ( Howard *et al.*, 2025). The jaAntXant3 sample was weighed and triaged to determine the appropriate extraction protocol. Tissue was homogenised by powermashing using a PowerMasher II tissue disruptor. HMW DNA was extracted using the Manual MagAttract v3 protocol. DNA was sheared into an average fragment size of 12–20 kb following the Megaruptor®3 for LI PacBio protocol. Sheared DNA was purified by automated SPRI (solid-phase reversible immobilisation). The concentration of the sheared and purified DNA was assessed using a NanoDrop spectrophotometer and Qubit Fluorometer using the Qubit dsDNA High Sensitivity Assay kit. Fragment size distribution was evaluated by running the sample on the FemtoPulse system. For this sample, the final post-shearing DNA had a Qubit concentration of 8.66 ng/μL and an average fragment size of 10.3 kb. The 260/280 spectrophotometric ratio was 1.78, and the 260/230 ratio was 1.63.

RNA was extracted from tentacle tissue of the jaAntXant4 specimen in the Tree of Life Laboratory at the WSI using the RNA Extraction: Automated MagMax™ *mir*Vana protocol. The RNA concentration was assessed using a NanoDrop spectrophotometer and a Qubit Fluorometer using the Qubit RNA Broad-Range Assay kit. Analysis of the integrity of the RNA was done using the Agilent RNA 6000 Pico Kit and Eukaryotic Total RNA assay.

### PacBio HiFi library preparation and sequencing

Library preparation and sequencing were performed at the WSI Scientific Operations core. Libraries were prepared using the SMRTbell Prep Kit 3.0 (Pacific Biosciences, California, USA), following the manufacturer’s instructions. The kit includes reagents for end repair/A-tailing, adapter ligation, post-ligation SMRTbell bead clean-up, and nuclease treatment. Size selection and clean-up were performed using diluted AMPure PB beads (Pacific Biosciences). DNA concentration was quantified using a Qubit Fluorometer v4.0 (Thermo Fisher Scientific) and the Qubit 1X dsDNA HS assay kit. Final library fragment size was assessed with the Agilent Femto Pulse Automated Pulsed Field CE Instrument (Agilent Technologies) using the gDNA 55 kb BAC analysis kit.

The sample was sequenced on a Revio instrument (Pacific Biosciences, California, USA). Prepared libraries with a molarity greater than 2 nM were normalised to 2 nM, and 15 μL was used for making complexes. For libraries with a molarity below 2 nM, the available 10 μL library volume was used without normalisation. Primers were annealed and polymerases were bound to generate circularised complexes, following the manufacturer’s instructions. Complexes were purified using 1.2× SMRTbell beads, diluted to the Revio loading concentration of 200–300 pM, and spiked with a Revio sequencing internal control. The sample was sequenced on a Revio 25 M SMRT cell. SMRT Link software (Pacific Biosciences), a web-based workflow manager, was used to configure and monitor the run and to carry out primary and secondary data analysis.

### Hi-C

***Sample preparation and crosslinking***

The Hi-C sample was prepared from 20–50 mg of frozen tissue from the jaAntXant4 tentacle using the Arima-HiC v2 kit (Arima Genomics). Following the manufacturer’s instructions, tissue was fixed and DNA crosslinked using TC buffer to a final formaldehyde concentration of 2%. The tissue was homogenised using the Diagnocine Power Masher-II. Crosslinked DNA was digested with a restriction enzyme master mix, biotinylated, and ligated. Clean-up was performed with SPRISelect beads before library preparation. DNA concentration was measured with the Qubit Fluorometer (Thermo Fisher Scientific) and Qubit HS Assay Kit. The biotinylation percentage was estimated using the Arima-HiC v2 QC beads.

***Hi-C library preparation and sequencing***

Biotinylated DNA constructs were fragmented using a Covaris E220 sonicator and size selected to 400–600 bp using SPRISelect beads. DNA was enriched with Arima-HiC v2 kit Enrichment beads. End repair, A-tailing, and adapter ligation were carried out with the NEBNext Ultra II DNA Library Prep Kit (New England Biolabs), following a modified protocol where library preparation occurs while DNA remains bound to the Enrichment beads. Library amplification was performed using KAPA HiFi HotStart mix and a custom Unique Dual Index (UDI) barcode set (Integrated DNA Technologies). Depending on sample concentration and biotinylation percentage determined at the crosslinking stage, libraries were amplified with 10–16 PCR cycles. Post-PCR clean-up was performed with SPRISelect beads. Libraries were quantified using the AccuClear Ultra High Sensitivity dsDNA Standards Assay Kit (Biotium) and a FLUOstar Omega plate reader (BMG Labtech).

Prior to sequencing, libraries were normalised to 10 ng/μL. Normalised libraries were quantified again to create equimolar and/or weighted 2.8 nM pools. Pool concentrations were checked using the Agilent 4200 TapeStation (Agilent) with High Sensitivity D500 reagents before sequencing. Sequencing was performed using paired-end 150 bp reads on the Illumina NovaSeq 6000.

### RNA library preparation and sequencing

Libraries were prepared using the NEBNext ^®^ Ultra II Directional RNA Library Prep Kit for Illumina (New England Biolabs), following the manufacturer’s instructions. Poly(A) mRNA was isolated from 100 ng of total RNA using oligo (dT) beads, converted to cDNA, and uniquely indexed; 14 PCR cycles were performed. Libraries were prepared with an intended average fragment size of 100–300 bp. Libraries were quantified, normalised, and pooled equimolarly to a final concentration of 2.8 nM before dilution to 150 pM for loading. Sequencing was carried out on the Illumina NovaSeq 6000, generating paired-end reads.

### Genome assembly

Prior to assembly of the PacBio HiFi reads, a database of *k*-mer counts ( *k* = 31) was generated from the filtered reads using FastK. GenomeScope2 ( Ranallo-Benavidez *et al.*, 2020) was used to analyse the *k*-mer frequency distributions, providing estimates of genome size, heterozygosity, and repeat content.

The HiFi reads were assembled using Hifiasm ( Cheng *et al.*, 2021) with the —primary option. Haplotypic duplications were identified and removed using purge_dups ( Guan *et al.*, 2020). The Hi-C reads ( Rao *et al.*, 2014) were mapped to the primary contigs using bwa-mem2 ( Vasimuddin *et al.*, 2019), and the contigs were scaffolded in YaHS ( Zhou *et al.*, 2023) with the —break option for handling potential misassemblies. The scaffolded assemblies were evaluated using Gfastats ( Formenti *et al.*, 2022), BUSCO ( Manni *et al.*, 2021) and MerquryFK ( Rhie *et al.*, 2020).

The mitochondrial genome was assembled using MitoHiFi ( Uliano-Silva *et al.*, 2023). The MitoHiFi reference was the mitochondrial genome of *Anthopleura anjunae* (NC_030274.1).

### Assembly curation

The assembly was decontaminated using the Assembly Screen for Cobionts and Contaminants ( ASCC) pipeline. TreeVal was used to generate the flat files and maps for use in curation. Manual curation was conducted primarily in PretextView and HiGlass ( Kerpedjiev *et al.*, 2018). Scaffolds were visually inspected and corrected as described by Howe *et al*. (2021). Manual corrections included 34 breaks, 60 joins, and removal of 14 haplotypic duplications. This reduced the scaffold count by 22.5%, increased the scaffold N50 by 6.7%, and reduced the total assembly length by 1.2%. The curation process is described at sanger-tol/curation-resources . PretextSnapshot was used to generate a Hi-C contact map of the final assembly.

### Assembly quality assessment

The Merqury.FK tool ( Rhie *et al.*, 2020) was run in a Singularity container ( Kurtzer *et al.*, 2017) to evaluate *k*-mer completeness and assembly quality for the primary and alternate haplotypes using the *k*-mer database ( *k* = 31) computed prior to genome assembly. The analysis outputs included assembly QV scores and completeness statistics.

The genome was analysed using the BlobToolKit pipeline, a Nextflow implementation of the earlier Snakemake version ( Challis *et al.*, 2020). PacBio reads were aligned to the assembly using minimap2 ( Li, 2018) and processed with SAMtools ( Danecek *et al.*, 2021) to generate coverage tracks. The pipeline runs BUSCO ( Manni *et al.*, 2021) using lineages identified from the NCBI Taxonomy ( Schoch *et al.*, 2020). For the three basal BUSCO lineages, eukaryota, bacteria and archaea, BUSCO genes are aligned to the UniProt Reference Proteomes database ( Bateman *et al.*, 2023) using DIAMOND BLASTp ( Buchfink *et al.*, 2021). The genome assembly is divided into chunks based on the density of BUSCO genes from the closest taxonomic lineage, and each chunk is aligned to the UniProt Reference Proteomes database using DIAMOND BLASTx. Sequences without DIAMOND BLASTx hits are extracted using seqtk and aligned to the NT database using BLASTn ( Altschul *et al.*, 1990). The outputs are consolidated into a BlobDir for visualisation in BlobToolKit. The BlobToolKit pipeline was developed using nf-core tooling ( Ewels *et al.*, 2020) and MultiQC ( Ewels *et al.*, 2016), with containerisation through Docker ( Merkel, 2014) and Singularity ( Kurtzer *et al.*, 2017).

## Genome sequence report

### Sequence data

**Figure 2.**
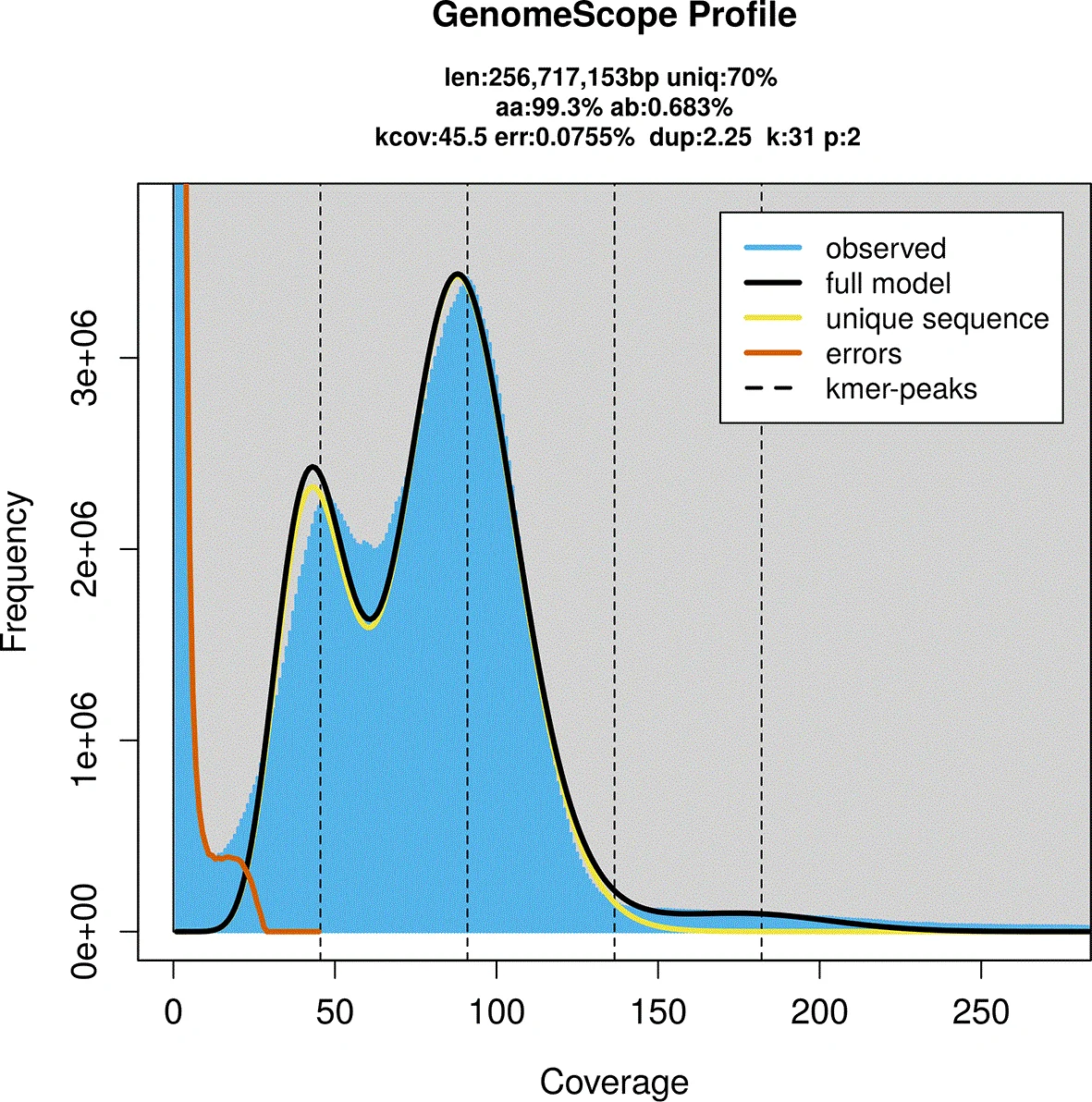
Frequency distribution of *k*-mers generated using GenomeScope2. The plot shows observed and modelled *k*-mer spectra, providing estimates of genome size, heterozygosity, and repeat content based on unassembled sequencing reads.

PacBio sequencing of the *Anthopleura xanthogrammica* specimen generated 24.02 Gb (gigabases) from 3.39 million reads, which were used to assemble the genome. GenomeScope2.0 analysis using a *p* = 2 model gave a *k*-mer-based genome size estimate of 257.22 Mb, with a heterozygosity of 0.68% and repeat content of 29.99% ( Figure 2). These estimates guided expectations for the assembly. Based on the estimated genome size, the sequencing data provided approximately 91× coverage. Hi-C sequencing produced 116.09 Gb from 384.41 million reads, which were used to scaffold the assembly. RNA sequencing data were also generated and are available in public sequence repositories. Table 1 summarises the specimen and sequencing details.

**Table 1.** Specimen and sequencing data for *Anthopleura xanthogrammica* (BioProject PRJEB73657).

Platform	PacBio HiFi	Hi-C	RNA-seq
**ToLID**	jaAntXant3	jaAntXant4	jaAntXant4
**Specimen ID**	UCALI0000033	UCALI0000035	UCALI0000035
**BioSample (source individual)**	SAMEA112465888	SAMEA112465890	SAMEA112465890
**BioSample (tissue)**	SAMEA112465893	SAMEA112465904	SAMEA112465902
**Tissue**	mid body	tentacle	tentacle
**Instrument**	Revio	Illumina NovaSeq 6000	Illumina NovaSeq 6000
**Run accessions**	ERR12736881	ERR12737277	ERR12737278
**Read count total**	3.39 million reads	384.41 million read pairs	23.38 million read pairs
**Base count total**	24.02 Gb	116.09 Gb	7.06 Gb

### Assembly statistics

The primary haplotype was assembled, and contigs corresponding to an alternate haplotype were also deposited in INSDC databases. The final assembly has a total length of 290.04 Mb in 133 scaffolds, with 176 gaps, and a scaffold N50 of 14.82 Mb ( Table 2).

**Table 2.** Genome assembly data for *Anthopleura xanthogrammica.*

Genome assembly	Primary assembly
**Assembly name**	jaAntXant3.1
**Assembly accession**	GCA_964019055.1
**Alternate haplotype accession**	GCA_964019075.1
**Assembly level**	chromosome
**Span (Mb)**	290.04
**Number of chromosomes**	19
**Number of contigs**	309
**Contig N50 (Mb)**	2.34
**Number of scaffolds**	133
**Scaffold N50 (Mb)**	14.82
**Organelles**	Mitochondrial genome: 20.04 kb

**Figure 3.**
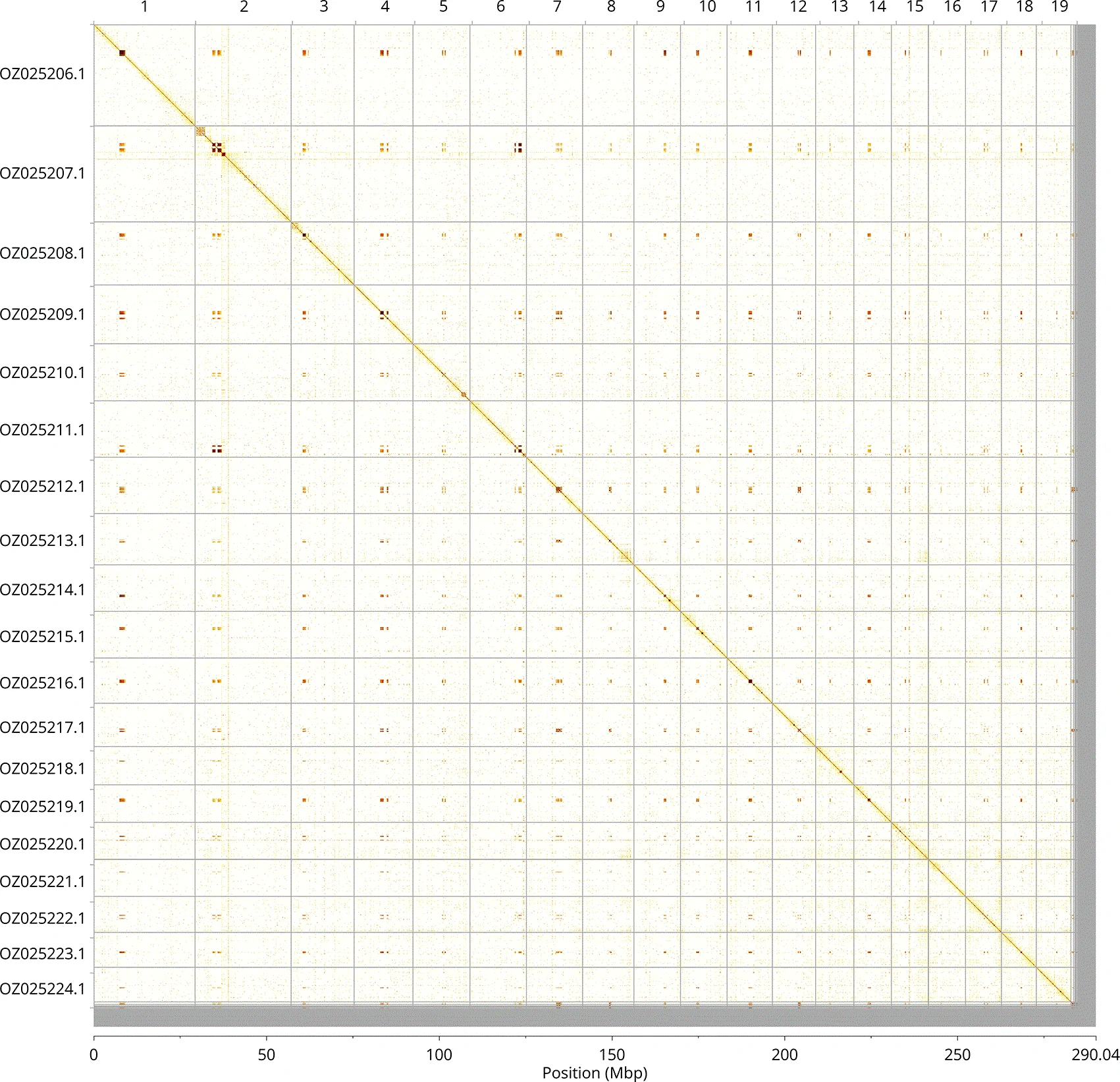
Hi-C contact map of the *Anthopleura xanthogrammica* genome assembly. Assembled chromosomes are shown in order of size and labelled along the axes, with a megabase scale shown below. The plot was generated using PretextSnapshot.

Most of the assembly sequence (97.36%) was assigned to 19 chromosomal-level scaffolds. These chromosome-level scaffolds, confirmed by Hi-C data, are named according to size ( Figure 3; Table 3).

**Table 3.** Chromosomal pseudomolecules in the primary genome assembly of *Anthopleura xanthogrammica* jaAntXant3.

INSDC accession	Molecule	Length (Mb)	GC%
OZ025206.1	1	29.29	37.50
OZ025207.1	2	27.74	37
OZ025208.1	3	18.19	37.50
OZ025209.1	4	17	37.50
OZ025210.1	5	16.47	37
OZ025211.1	6	16.35	37
OZ025212.1	7	16.17	38
OZ025213.1	8	14.82	37.50
OZ025214.1	9	13.52	37
OZ025215.1	10	13.43	37.50
OZ025216.1	11	13.06	37.50
OZ025217.1	12	12.51	37.50
OZ025218.1	13	11.08	37.50
OZ025219.1	14	10.84	37
OZ025220.1	15	10.72	37
OZ025221.1	16	10.68	37.50
OZ025222.1	17	10.38	37.50
OZ025223.1	18	10.08	37.50
OZ025224.1	19	10.03	37.50

The mitochondrial genome was also assembled (length 20.04 kb, OZ025225.1). This sequence is included as a contig in the multifasta file of the genome submission and as a standalone record.

### Assembly quality metrics

**Figure 4.**
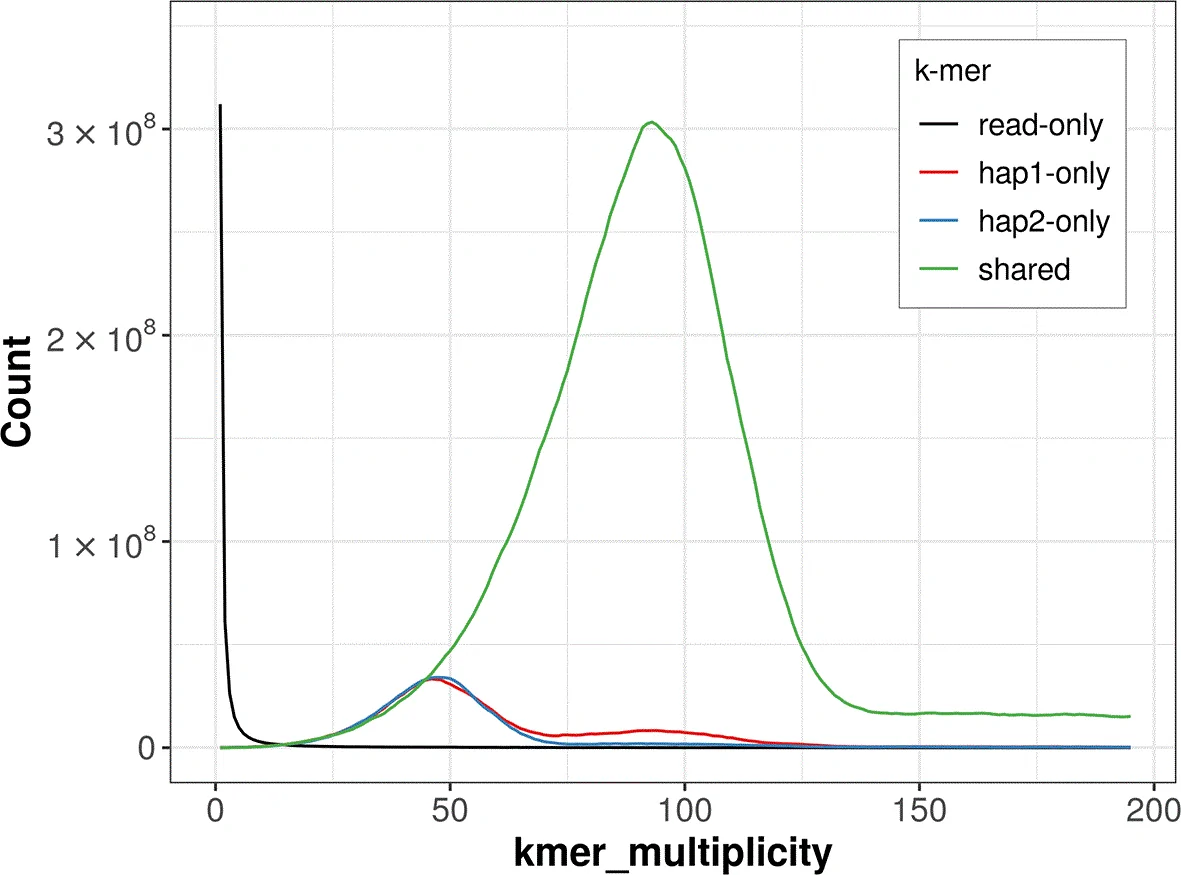
Evaluation of *k*-mer completeness using MerquryFK. This plot illustrates the recovery of *k*-mers from the original read data in the final assemblies. The horizontal axis represents *k*-mer multiplicity, and the vertical axis shows the number of *k*-mers. The black curve represents *k*-mers that appear in the reads but are not assembled. The green curve corresponds to *k*-mers shared by both haplotypes, and the red and blue curves show *k*-mers found only in one of the haplotypes.

The combined primary and alternate assemblies achieve an estimated QV of 54.3. The *k*-mer completeness is 89.92% for the primary assembly, 88.80% for the alternate haplotype, and 99.58% for the combined assemblies ( Figure 4).

**Figure 5.**
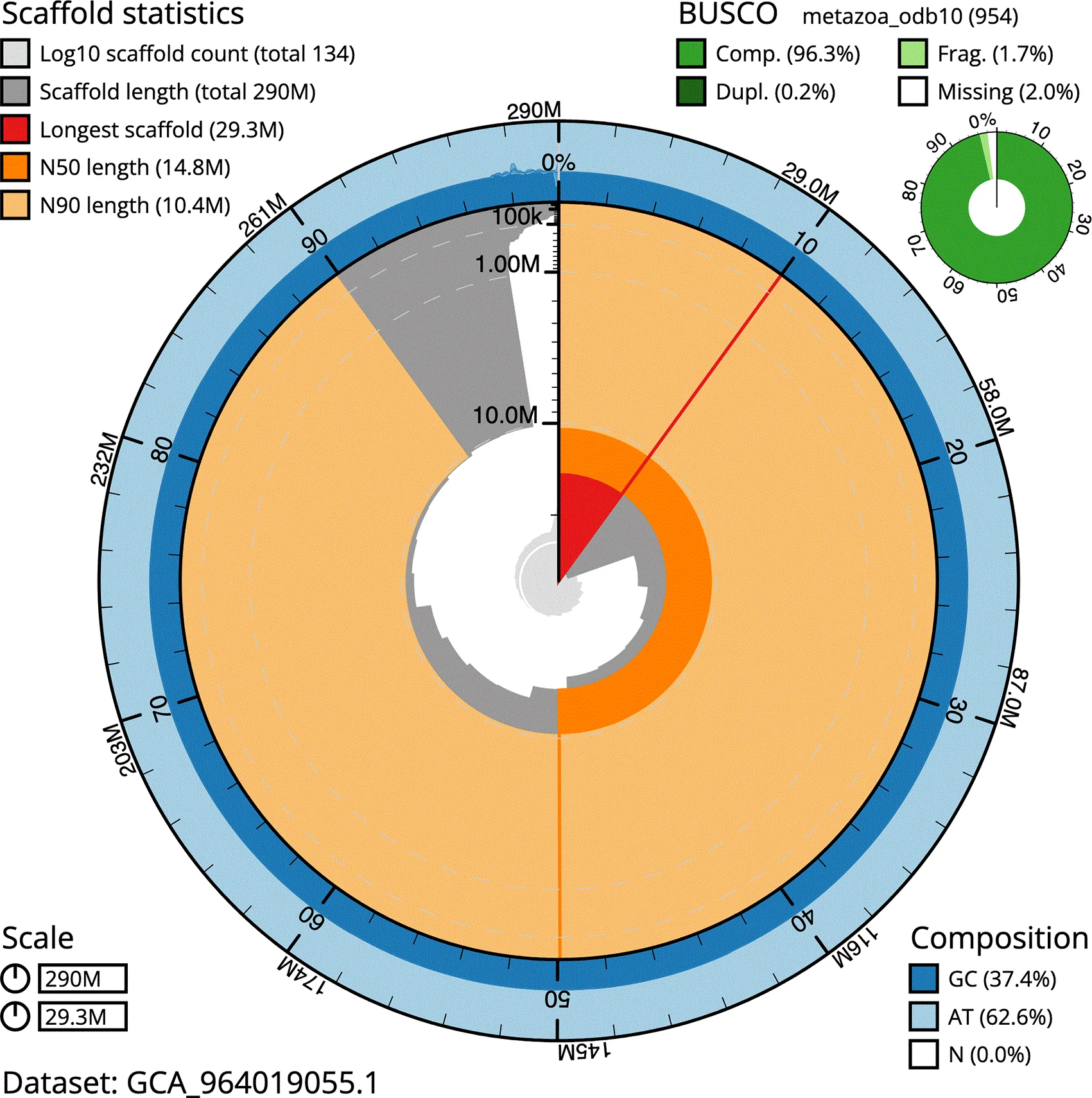
Assembly metrics for jaAntXant3.1. The BlobToolKit snail plot provides an overview of assembly metrics and BUSCO gene completeness. The circumference represents the length of the whole genome sequence, and the main plot is divided into 1 000 bins around the circumference. The outermost blue tracks display the distribution of GC, AT, and N percentages across the bins. Scaffolds are arranged clockwise from longest to shortest and are depicted in dark grey. The longest scaffold is indicated by the red arc, and the deeper orange and pale orange arcs represent the N50 and N90 lengths. A light grey spiral at the centre shows the cumulative scaffold count on a logarithmic scale. A summary of complete, fragmented, duplicated, and missing BUSCO genes in the metazoa_odb10 set is presented at the top right. An interactive version of this figure can be accessed on the BlobToolKit viewer.

**Figure 6.**
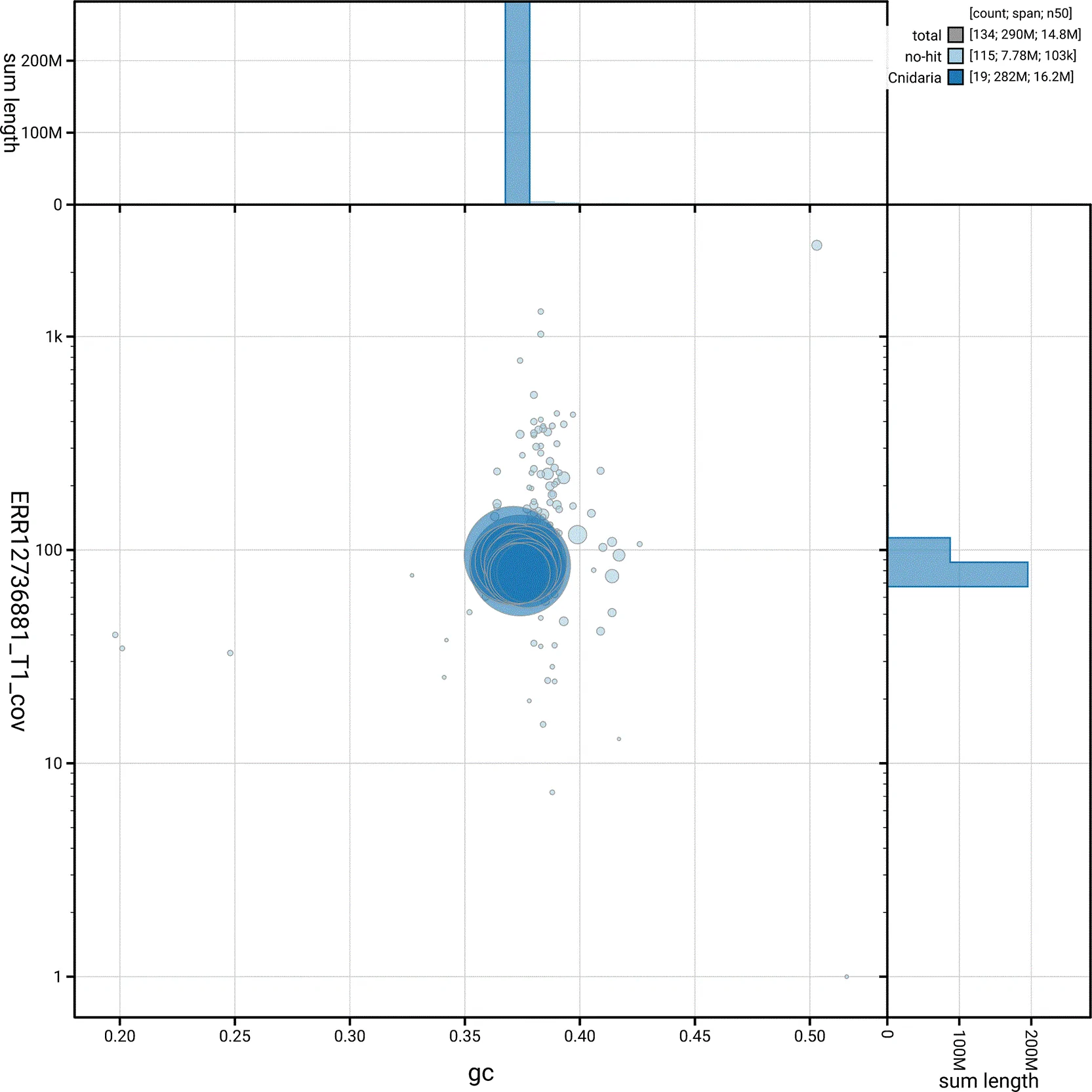
BlobToolKit blob plot for jaAntXant3.1. The plot shows base coverage (vertical axis) and GC content (horizontal axis). The circles represent scaffolds, with the size proportional to scaffold length and the colour representing phylum membership. The histograms along the axes display the total length of sequences distributed across different levels of coverage and GC content. An interactive version of this figure is available on the BlobToolKit viewer.

BUSCO v.5.5.0 analysis using the metazoa_odb10 reference set ( *n* = 954) identified 96.3% of the expected gene set (single = 96.1%, duplicated = 0.2%). The snail plot in Figure 5 summarises the scaffold length distribution and other assembly statistics for the primary assembly. The blob plot in Figure 6 shows the distribution of scaffolds by GC proportion and coverage.

Table 4 lists the assembly metric benchmarks adapted from Rhie *et al.* (2021) and the Earth BioGenome Project (EBP) Report on Assembly Standards January 2026. The EBP metric, calculated for primary assembly, is **6.C.Q54**, meeting the recommended 6.C.Q40 reference standard.

**Table 4.** Earth Biogenome Project summary metrics for the *Anthopleura xanthogrammica* assembly.

Measure	Value	Benchmark
EBP summary (primary)	6.C.Q54	6.C.Q40
Contig N50 length	2.34 Mb	≥ 1 Mb
Scaffold N50 length	14.82 Mb	= chromosome N50
Consensus quality (QV)	Primary: 54.6; alternate: 54.2; combined: 54.3	≥ 40
*k*-mer completeness	Primary: 89.92%; alternate: 88.80%; combined: 99.58%	> 90%
BUSCO	C:96.3% [S:96.1%, D:0.2%], F:1.7%, M:2.0%, n:954	S > 90%; D < 5%
Percentage of assembly assigned to chromosomes	97.36%	≥ 90%

*Notes:* The EBP summary uses log10(Contig N50); chromosome-level (C) or log10(Scaffold N50); Q (Merqury QV). BUSCO: C = complete; S = single-copy; D = duplicated; F = fragmented; M = missing; n = orthologues

**Table 5.** Software versions and sources used for *Anthopleura xanthogrammica.*

Software	Version	Source
BLAST	2.14.0	https://ftp.ncbi.nlm.nih.gov/blast/executables/blast+/
blobtk	0.5.1	https://github.com/genomehubs/blobtk
BlobToolKit	4.3.9	https://github.com/genomehubs/blobtoolkit
BUSCO	5.5.0; 6.1.0	https://gitlab.com/ezlab/busco
bwa-mem2	2.2.1	https://github.com/bwa-mem2/bwa-mem2
DIAMOND	2.1.8	https://github.com/bbuchfink/diamond
fasta_windows	0.2.4	https://github.com/tolkit/fasta_windows
FastK	1.1	https://github.com/thegenemyers/FASTK
GenomeScope2.0	2.0.1	https://github.com/tbenavi1/genomescope2.0
Gfastats	1.3.6	https://github.com/vgl-hub/gfastats
Hifiasm	0.19.8-r603	https://github.com/chhylp123/hifiasm
HiGlass	1.13.4	https://github.com/higlass/higlass
MerquryFK	1.1.0-c1	https://github.com/thegenemyers/MERQURY.FK
Minimap2	2.24-r1122	https://github.com/lh3/minimap2
MitoHiFi	3	https://github.com/marcelauliano/MitoHiFi
MultiQC	1.14; 1.17 and 1.18	https://github.com/MultiQC/MultiQC
Nextflow	23.04.1; 23.10.0; 25.10.0; 25.10.4	https://github.com/nextflow-io/nextflow
PretextSnapshot	0.0.6	https://github.com/sanger-tol/PretextSnapshot
PretextView	1.0.3	https://github.com/sanger-tol/PretextView
purge_dups	1.2.3	https://github.com/dfguan/purge_dups
samtools	1.16.1; 1.17; 1.18; 1.6	https://github.com/samtools/samtools
sanger-tol/ascc	0.1.0	https://github.com/sanger-tol/ascc
sanger-tol/blobtoolkit	0.4.0	https://github.com/sanger-tol/blobtoolkit
sanger-tol/curationpretext	1.4.2	https://github.com/sanger-tol/curationpretext
Seqtk	1.4-r122	https://github.com/lh3/seqtk
Singularity	3.9.0	https://github.com/sylabs/singularity
TreeVal	1.0.2	https://github.com/sanger-tol/treeval
YaHS	1.1a.2	https://github.com/c-zhou/yahs

### Genome annotation report

The *Anthopleura xanthogrammica* genome assembly (GCA_964019055.1) was annotated by Ensembl at the European Bioinformatics Institute (EBI). This annotation includes 50,518 transcribed mRNAs from 25,034 protein-coding and 9,688 non-coding genes. The average transcript length is 6,792.22 bp, with an average of 1.45 coding transcripts per gene and 6.96 exons per transcript. Further details of this annotation are available from the Ensembl annotation page.

## Author information

Contributors are listed at the following links:

• Members of the Wellcome Sanger Institute Tree of Life Management, Samples and Laboratory team
• Members of Wellcome Sanger Institute Scientific Operations – Sequencing Operations
• Members of the Wellcome Sanger Institute Tree of Life Core Informatics team
• Members of the EBI Aquatic Symbiosis Genomics Data Portal Team
• The Aquatic Symbiosis Genomics Project leadership

## Wellcome sanger institute – legal and governance

The materials that have contributed to this genome note have been supplied by a Tree of Life collaborator. The Wellcome Sanger Institute employs a process whereby due diligence is carried out proportionate to the nature of the materials themselves, and the circumstances under which they have been/are to be collected and provided for use. The purpose of this is to address and mitigate any potential legal and/or ethical implications of receipt and use of the materials as part of the research project, and to ensure that in doing so we align with best practice wherever possible.

The overarching areas of consideration are:

• Ethical review of provenance and sourcing of the material
• Legality of collection, transfer and use (national and international)

Each transfer of samples is undertaken according to a Research Collaboration Agreement or Material Transfer Agreement entered into by the Tree of Life collaborator, Genome Research Limited (operating as the Wellcome Sanger Institute) and in some circumstances other Tree of Life collaborators.

## Data Availability

European Nucleotide Archive: Anthopleura xanthogrammica (giant green sea anemone). Accession number PRJEB73657; https://identifiers.org/ena.embl/PRJEB73657. The genome sequence is released openly for reuse. The *Anthopleura xanthogrammica* genome sequencing initiative is part of the Aquatic Symbiosis Genomics Project ( PRJEB43743) and the Sanger Institute Tree of Life Programme ( PRJEB43745). All raw sequence data and the assembly have been deposited in INSDC databases. Raw data and assembly accession identifiers are reported in Tables 1 and 2. Production code used in genome assembly at the WSI Tree of Life is available at https://github.com/sanger-tol . Table 5 lists software versions used in this study.
