# Supplementary material for: The chromosomal genome sequence of the giant green anemone, Anthopleura xanthogrammica (Brandt, 1835) (Actiniaria: Actiniidae)
Source: Wellcome Open Res. 2026 Sep 1;11:566. [Version 1] doi: 10.12688/wellcomeopenres.27544.1 (PMC13598401; doi:10.12688/wellcomeopenres.27544.1)
Supplement: Peer review history

# Reviewer report: Reviewer response for version 1

**DOI:** 10.21956/wellcomeopenres.30317.r167245

**Published:** 2026-09-22

**Authors:** Adrián Jaimes-Becerra

**Affiliations:** 1 Tohoku University, Sendai, Japan

This article presents a chromosome-scale nuclear genome and mitochondrial genome for the giant green sea anemone *Anthopleura xanthogrammica*. PacBio HiFi reads were generated from specimen jaAntXant3 collected at Seal Rock, Oregon. Hi-C and RNA-seq reads came from a second specimen, jaAntXant4, collected at Hopkins Marine Station, California. The final primary assembly spans 290.04 Mb in 133 nuclear scaffolds. The reported consensus QV is 54.6 for the primary assembly, BUSCO completeness is 96.3% using metazoa_odb10, and the combined primary-plus-alternate k-mer completeness is 99.58%. The mitochondrial sequence is 20.04 kb, and Ensembl reports 25,034 protein-coding genes. The principal contribution is a high-contiguity, publicly released reference genome for an ecologically important, photosymbiotic actiniarian. It should support comparative work on symbiosis, population biology, chromosome evolution, and environmental responses in Anthopleura. This is a valuable resource contribution.

 The article is strong, and the assembly statistics are consistent with a useful chromosome-scale genome. The writing is generally clear, the figures are legible, and the article is appropriate in scope for a data note. The manuscript also provides public raw-read and assembly accessions and uses well-established assembly and quality-control tools.

 I have only a few minor comments:

 1. The PacBio library-preparation section states that “size selection and clean-up were performed using diluted AMPure PB beads,” but neither the bead dilution nor the bead-to-sample volume ratio is provided. Please report these details, if possible.

 2. The manuscript states that PacBio sequencing generated 24.02 Gb from 3.39 million reads and subsequently reports approximately 91X genome coverage. Please clarify whether the 24.02 Gb represents the raw sequencing yield or the amount remaining after read filtering. 

 3. ASCC decontamination is described after scaffolding, but the manuscript does not report the outcome of this step. 

 4. The Fautin and Hand reference and its associated link should be checked. The DOI currently provided (`https://doi.org/10.17161/1808.32247`) does not resolve to the cited work.

Are sufficient details of methods and materials provided to allow replication by others?

Yes

Is the rationale for creating the dataset(s) clearly described?

Yes

Are the datasets clearly presented in a useable and accessible format?

Yes

Are the protocols appropriate and is the work technically sound?

Yes

Reviewer Expertise:

Evolutionary biology, Cnidaria, Bioinformatics, Genomics.

I confirm that I have read this submission and believe that I have an appropriate level of expertise to confirm that it is of an acceptable scientific standard.

# Reviewer report: Reviewer response for version 1

**DOI:** 10.21956/wellcomeopenres.30317.r167250

**Published:** 2026-09-22

**Authors:** Wai Lok So

**Affiliations:** 1 The Hang Seng University of Hong Kong, Hong Kong, Hong Kong

The manuscript presents a high-quality genome assembly of *Anthopleura xanthogrammica*. The background is well-written with sufficient details about the species. The sequencing and assembly methods are technically sound and standardised, yielding a chromosomal-level genome, together with a complete mitochondrial genome. 

 A few minor comments as follows:

 1. There is an inconsistency between the use of "giant green anemone" in abstract and keywords, and "giant green sea anemone" in background. Please kindly make corrections.

 2. Please kindly add a scale bar on Figure 1 to indicate the size of the sea anemone.

 3. Is there a procedure in removing the symbiotic algae in the anemone before getting the tissue for sequencing. If so, please kindly state the procedure.

Are sufficient details of methods and materials provided to allow replication by others?

Yes

Is the rationale for creating the dataset(s) clearly described?

Yes

Are the datasets clearly presented in a useable and accessible format?

Yes

Are the protocols appropriate and is the work technically sound?

Yes

Reviewer Expertise:

evolution, genomics, arthropods, myriapods

I confirm that I have read this submission and believe that I have an appropriate level of expertise to confirm that it is of an acceptable scientific standard.

# Reviewer report: Reviewer response for version 1

**DOI:** 10.21956/wellcomeopenres.30317.r167248

**Published:** 2026-09-17

**Authors:** Abdelmalek Lekired

**Affiliations:** 1 University of New Mexico, Albuquerque, New Mexico, USA

The chromosomal genome sequence of the giant green anemone, *Anthopleura xanthogrammica* (Brandt, 1835) (Actiniaria: Actiniidae)

 Comments

 Figure 1: Please mention the full name of the region from where the sampling was done.

 Hifiasm could purge duplicates internally why you did not used it ?

 Why you remove contaminant after scaffolding ?

 How did you identify contaminants if they were incorporated in the chromosomes?

 Why you did not mention the BUSCO results for the primary assembly and for the final genome ?

 This paragraph is not clear

 “BUSCO v.5.5.0 analysis using the metazoa_odb10 reference set (n = 954) identified 96.3% of the expected gene set (single = 96.1%, duplicated = 0.2%). The snail plot in Figure 5 summarises the scaffold length distribution and other assembly statistics for the primary assembly. The blob plot in Figure 6 shows the distribution of scaffolds by GC proportion and coverage.”

 The busco results is for the primary assembly or the scaffolded assembly after removing contaminants??

 The sum of 19 chromosome is only 282.36 Mb but the snail plot shows 290 Mb ??

Are sufficient details of methods and materials provided to allow replication by others?

No

Is the rationale for creating the dataset(s) clearly described?

Yes

Are the datasets clearly presented in a useable and accessible format?

Partly

Are the protocols appropriate and is the work technically sound?

No

Reviewer Expertise:

Genomics, annotation, RNAseq, Bacterial genomics, fungal genomics, invertebrate genomics.

I confirm that I have read this submission and believe that I have an appropriate level of expertise to confirm that it is of an acceptable scientific standard, however I have significant reservations, as outlined above.

# Reviewer report: Reviewer response for version 1

**DOI:** 10.21956/wellcomeopenres.30317.r167251

**Published:** 2026-09-14

**Authors:** Bingpeng Xing

**Affiliations:** 1 Third Institute of Oceanography Ministry of Natural Resources, Xiamen, Fujian, China

The PacBio HiFi data were generated from specimen jaAntXant3 collected in Oregon, whereas the Hi-C and RNA-seq data were obtained from jaAntXant4 collected in California. Because these specimens originated from geographically distant populations, the authors should clarify how both individuals were identified as A. xanthogrammica and whether molecular barcodes or other genetic evidence confirmed their conspecificity. The possible influence of using Hi-C reads from a different individual for scaffolding should also be briefly discussed.

 The estimated genome size based on k-mers was 257.22 Mb, whereas the final primary assembly spans 290.04 Mb, approximately 13% larger. Please briefly explain this discrepancy and clarify whether residual haplotypic duplication, biological variation, or limitations of the k-mer estimate may have contributed.

Are sufficient details of methods and materials provided to allow replication by others?

Yes

Is the rationale for creating the dataset(s) clearly described?

Yes

Are the datasets clearly presented in a useable and accessible format?

Yes

Are the protocols appropriate and is the work technically sound?

Yes

Reviewer Expertise:

genome，marine biology，biodiversity

I confirm that I have read this submission and believe that I have an appropriate level of expertise to confirm that it is of an acceptable scientific standard.
